# A clinically applicable molecular classification for high-grade serous ovarian cancer based on hormone receptor expression

**DOI:** 10.1038/srep25408

**Published:** 2016-05-03

**Authors:** Zheng Feng, Hao Wen, Rui Bi, Xingzhu Ju, Xiaojun Chen, Wentao Yang, Xiaohua Wu

**Affiliations:** 1 Department of Gynecological Oncology, Fudan University Shanghai Cancer Center, Shanghai 200032, China; 2 Department of Oncology, Shanghai Medical College, Fudan University, Shanghai 200032, China; 3 Department of Pathology, Fudan University Shanghai Cancer Center, Shanghai 200032, China

## Abstract

To establish an effective hormone receptor-based molecular classification of high-grade serous ovarian cancer (HGSC), we retrospectively examined 875 consecutive HGSC patients who underwent primary surgery at our hospital and constructed tissue microarrays from these specimens. The expression levels of the hormone receptors were as follows: ER 64.4%, PR 12.6%, AR 35.6%, FSHR 54.5%, LHR 34.8%, and GnRHR 88.3%. Based on clustering of their expression patterns, we classified patients into five subgroups with distinctive clinical features (PR+, PR − ER + AR+, PR − ER + AR−, PR − ER − AR+, and PR − ER − AR−). Patients in the PR + group were younger compared to those in the other groups (p < 0.001). More patients were of advanced stage in the PR − ER + AR− group than the other groups (p = 0.020). A greater proportion of patients were sensitive to platinum-based chemotherapy in the PR − ER − AR + group compared with the other groups (p = 0.034). A trend of increasing risk of death was observed among these subgroups (p < 0.001). In the multivariate analysis, patients also had orderly increased hazard ratios for death in the PR + (HR = 2.256, 95% CI, 0.983–5.175), PR − ER + AR + (HR = 2.188, 95% CI, 1.004–4.796), PR − ER − AR− (HR = 2.316, 95% CI, 1.097–5.082) and PR − ER + AR− (HR = 2.928, 95% CI, 1.366–6.276) subgroups compared to the PR − ER − AR+ subgroup. Our classification could help predict patient clinical outcomes, guide individual treatments and stratify patients in future clinical trials.

Ovarian cancer is the seventh most commonly diagnosed disease worldwide as well as the eighth most lethal disease among females around the world^1^. After primary treatment including staging or debulking surgery and platinum-based adjuvant chemotherapy, around half of patients will relapse within 16 months^2^. Thus, effective clinic-pathological biomarkers are urgently required.

Epidemiological studies have indicated the potential role of steroid hormone in the etiology of ovarian cancer^2^. Hormone receptors, including estrogen receptor (ER), progesterone receptor (PR), androgen receptor (AR), follicle-stimulating hormone receptor (FSHR), luteinizing hormone receptor (LHR) and gonadotropin-releasing receptor (GnRHR), could mediate the effects of steroid hormones on ovarian cancer development and progression^3^,^4^,^5^,^6^,^7^. Previous studies have shown that ER and PR expression could be prognostic biomarkers of ovarian cancer. However, these results are inconsistent and sometimes contradictory^8^,^9^,^10^,^11^,^12^. Studies that describe the associations between AR, FSH-R, LH-R, and GnRHR expression and ovarian cancer survival are relatively sparse^7^,^12^,^13^,^14^,^15^.

In addition, epithelial ovarian cancers are a group of heterogeneous tumors based on distinctive morphological and molecular genetic features^16^. As mentioned above, most studies combined all of the disease subtypes and had small sample sizes. This may hinder efforts to identify the subtype-specific significance of hormone receptor expression. Some conflicting data are difficult to interpret^8^,^9^,^10^,^11^. Additionally, because the vast majority of ovarian cancers are HGSCs, meaningful and reliable indicators for further classifying this large group of patients are needed.

We analyzed the expression levels of hormone receptors according to the hypothalamic-pituitary-gonadal axis and another two potentially useful biomarkers (HER2 and Ki67) in 875 patients with HGSC. A new hormone receptor-based classification of HGSC was established, and patients were divided into five subgroups with distinctive clinical features.

## Methods

### Clinical Data

The clinical data were collected retrospectively from women who underwent primary surgery for HGSC at our hospital between April, 2005 and June, 2013. This study was conducted according to the Declaration of Helsinki and was approved by the Committee at Fudan University Shanghai Cancer Center. All participants provided written informed consent. Patients were excluded if they had received neoadjuvant chemo therapy, had been treated for recurrent disease, or were found to have other histological diagnoses on pathological review.

Clinical and pathological data were obtained from the medical records, cancer registries, and pathology reports. Patient characteristics, including age, menopausal status, FIGO stage, surgical outcomes, date of progression or recurrence, and the patient’s disease status at last contact, were collected. All patients were followed up until December 31, 2014.

R0 was defined as no macroscopic residual disease (RD) after surgery. Platinum sensitivity was defined as a time interval of 6 months or longer between the completion of platinum-based chemotherapy and the detection of relapse. Platinum resistance was defined as disease progression during adjuvant chemotherapy or within the 6-month interval between the completion of chemotherapy and disease relapse.

Progression-free survival (PFS) was defined as the time interval from the date of primary surgery to the date of disease progression or recurrence. Overall survival (OS) was defined as the time interval from the date of the primary surgery to the date of death or the last follow-up.

### Tissue Microarray and Immunohistochemistry

The histological diagnoses were based on the WHO criteria^17^. The samples were re-reviewed and reclassified as low and high-grade serous carcinoma based on the two-tiered grading system by two experienced gynecological pathologists (two co-authors of this paper). A microarray (1 mm) with triplicate tissue samples from each tumor was prepared^9^,^18^. Immunohistochemical staining was performed for ER, PR, HER2 and Ki-67 using a Ventana Benchmark XT autostainer (Ventana Medical Systems Inc., Tucson, AZ, USA). Staining for AR, FSHR, LHR and GnRHR was performed using the Envision horseradish peroxidase system (DAKO EnVision System K5007) following the manufacturer’s protocol. The following primary antibodies were used: ER (Roche SP1), PR (Roche 1E2), AR (Abcam ab133273, 1:100), FSH-R (Abcam ab150557, 1:100), LH-R (Santa Cruz sc-25828, 1:40), GnRH-R (Abcam ab183079, 1:50), HER2 (Roche 4B5), and Ki67 (Roche 30–9).

The results were independently judged, evaluated, and scored by two experienced gynecological pathologists without knowledge of the patients’ information. The results were recorded as the numerical mean of the values obtained from the triplicate cores. The intra-class correlation coefficient (ICC) was calculated to evaluate the internal consistency of the immunoscore of the three cores from each individual tumor sample. The Cronbach’s α indexes were approximately 0.9, which meant that there were no differences in parameter expression among the different morphological tissues. The expression levels of hormone receptors were determined using the following criteria:

ER, PR and AR levels: >10% showing positive nuclear staining of any intensity was defined as positive^19^,^20^.

FSH-R and LH-R levels: Evaluation of the cytoplasmic staining reaction was performed in accordance with the immunoreactive score (IRS). The IRS was defined as staining intensity (SI) by the percentage of positive cells (PP). SI was defined as 0 (negative), 1 (weak), 2 (moderate) and 3 (strong). PP was defined as 0 (negative), 1 (no more than 10% positive cells), 2 (11% to 50% positive cells), 3 (51% to 80% positive cells) and 4 (more than 80% positive cells). IRS = SI × PP, IRS ≥ 3 was defined as positive^21^.

GnRHR level: The cytoplasmic staining of GnRHR was recorded as negative, weak, moderate and strong. Staining of any intensity was regarded as positive^22^.

HER2 level: Membrane HER2 staining was recorded by scores of 0, 1+, 2+ and 3+ according to the ASCO/CAP guideline^23^. In our cohort, any score of >0 (1+, 2+, and 3+), not only 3+, was regarded as positive.

Ki67 level: >50% showing positive nuclear staining of any intensity was defined as positive, which could discriminate patients into groups with different prognoses^24^.

### Statistical Analyses

SPSS software (version 21.0, IBM Inc, USA), R software (version 3.2, Mathsoft Inc, USA) and GraphPad Prism software (version 6.0, GraphPad software Inc, USA) were used for the statistical analyses. Descriptive statistics were summarized as the means with the standard deviations (SD), the medians with the interquartile ranges (IQRs) or ranges, or the frequencies with the percentages. The categorical data were compared with chi-square or Fisher’s exact tests as appropriate. Logistic regression analysis was used in the multivariate analyses to evaluate the effects of the predictive factors, which are expressed as odds ratios (ORs). After the predictive and prognostic analyses, we identified ER, PR, AR and Ki67 as meaningful clinical indicators for HGSC molecular classification. An unsupervised hierarchical clustering analysis was performed to identify which tumors were related to each other according to their expression regardless of other patient characteristics. And average linkage clustering was used based on the positive and negative expression values. The PFS and OS were analyzed with the Kaplan-Meier method and log-rank tests in the univariate analyses, and cox regression analysis was used in the multivariate analyses to evaluate the effects of the prognostic factors, which are expressed as hazard ratios (HRs). All patients were included in the OS analyses, however, 72 patients (8.2%) with missing data on recurrence were excluded from the PFS analyses. Among them, 24 patients could not recall their exact recurrence date, and 48 patients were recorded as died from cancer recurrence in the cancer registries but their relapse dates were not documented. P < 0.05 was considered statistically significant, and all reported P values were 2-sided.

## Results

### Patient characteristics and hormone receptor expression levels

The patient characteristics and hormone receptor expression levels are described in Table 1. Among the 875 patients, 602 of them (69%) were postmenopausal and 85 of them (10%) had a family history of breast or ovarian cancer. A total of 800 patients (91%) were of advanced stage (III–IV).

Because some cores in the TMA slides shed off during the procedure of IHC staining, only 863–868 patients had available expression data for each IHC parameter. Representative images of parameter staining are shown in Figure S1. These parameters were present in the majority of HGSC patients. A total of 556 (64%) patients were ER positive. PR and AR were highly expressed in 13% and 36% of patients, respectively. Nearly 90% of patients were GnRHR positive; however, for HER2 staining, only 4% of patients were scored ≥1 (score 1+: 26 patients; score 2+: 3 patients; and score 3+: 2 patients, respectively). FSHR, LHR and Ki67 were highly expressed in 55%, 35% and 26% of the patients, respectively. The associations of these parameters are shown in Table S1.

### Independent analyses of hormone receptor expression levels

All 875 patients in our study underwent primary staging or debulking surgery, and 272 (31%) of them were debulked to R0 after primary surgery. We did not observe any associations between residual disease and hormone receptor expression or expression of any other parameters (Table S2).

A total of 849 (97%) patients had received platinum-based adjuvant chemotherapy following primary surgery. The majority of patients were administered taxanes (including paclitaxel (646/875, 74%), docetaxel (29/875, 8%)). A small number of patients received cyclophosphamide (18/875, 2%). Information regarding the combination of agents received by 23% (200/875) of the patients was not available. A total of 568 (67%) patients were platinum sensitive (Table 1). A greater proportion of the PR-positive (80.4% *vs.* 69.1%, p = 0.020) or AR-positive (76.2% *vs.* 67.3%, p = 0.010) patients were sensitive to platinum-based chemotherapy compared to the corresponding negative patients. Moreover, a larger proportion of patients with Ki67 over 50% were platinum sensitive compared to patients with Ki67 below 50% (Table 2). We did not observe any associations between platinum sensitivity and ER, FSHR, LHR, GnRHR or HER2 expression (Table 2). In the multivariate analysis, AR expression (OR = 0.625, 0.434–0.900, p = 0.011) and Ki67 over 50% (OR = 0.632, 0.429–0.931, p = 0.020) were independent predictors of platinum sensitivity (Table S3).

The median follow-up time was 29 (1–115) months. A total of 499 (57.0%) patients had documented recurrence with a median (95% CI) PFS of 18 (16.8–19.2) months. The 2-year and 5-year PFSs were 38.1% and 19.4%, respectively. Among all of the patients in the study, 345 (39.4%) deaths were documented, and the median (95% CI) OS was 58 (51.4–64.6) months. The 2-year and 5-year OSs were 79.3% and 48.8%, respectively. The known negative influences on PFS or OS were confirmed and included advanced FIGO stage (p < 0.001 and < 0.001, respectively) and the presence of residual disease (p < 0.001 and <0.001, respectively).

In the univariate analyses of PFS, ER expression was associated with impaired PFS, while PR expression was associated with improved PFS (p = 0.036 and 0.009, respectively, Figure S2, Table 3). The women with Ki67 over 50% tended to exhibit longer PFS than those with Ki67 below 50% (p = 0.021, Figure S2, Table 3). The associations between AR, FSHR, LHR GnRHR or HER2 expression and PFS revealed no statistically significant differences. In the multivariate analysis with adjustments for age, FIGO stage, and cytoreduction outcome, ER (HR = 1.302, 1.077–1.573, p = 0.006) and PR (HR = 0.718, 0.538–0.958, p = 0.024) expression were found to be independent predictors of PFS (Table 3).

In the univariate analyses of OS, AR expression and Ki67 over 50% were associated with improved OS (p = 0.023 and 0.003, Figure S3, Table 3). The women with positive PR expression tended to exhibit longer OS than those with negative PR expression; however, the difference was not significant (p = 0.061, Figure S3, Table 3). The associations between FSHR, LHR, GnRHR or HER2 expression and OS revealed no statistically significant differences. In the multivariate analysis with adjustments for age, FIGO stage, and cytoreduction outcome, ER (HR = 1.288, 1.014–1.636, p = 0.038), AR (HR = 0.744, 0.578–0.959, p = 0.022) and Ki67 (HR = 0.688, 0.520–0.910, p = 0.009) expression were found to be independent predictors of OS. PR expression was no longer an independent predictor of OS (HR = 0.911, 0.617–1.346, p = 0.641, Table 3).

### Molecular subtype classification of HGSC

**Figure 1:**
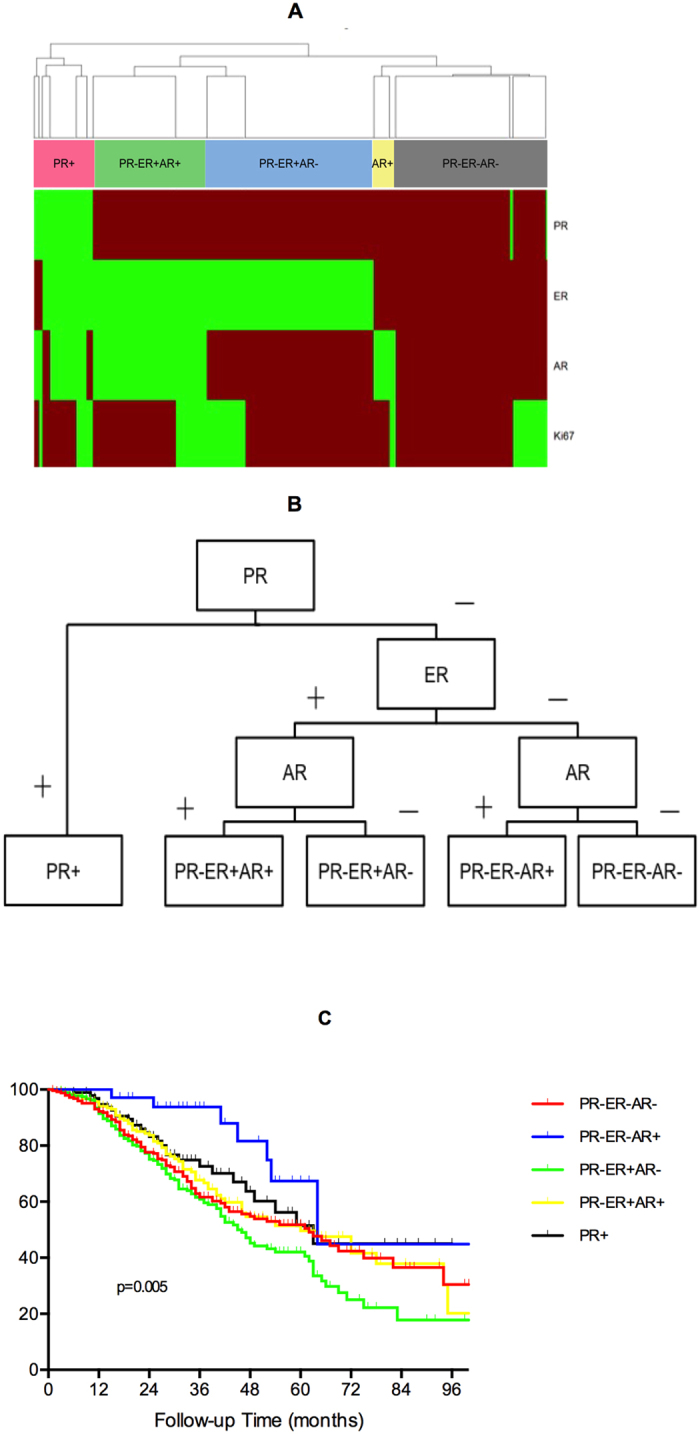
Molecular classification of patients based on clustering analysis of ER, PR, AR and Ki67 expression. (**A**) Clustering analysis divided the patients into five subgroups. Horizontally, the expression for the different receptors is depicted, with green indicating positive expression and red indicating negative expression. (**B**) Algorithm for the classification of the five subgroups. (**C**) Kaplan-Meier curve of OS stratified by the five subgroups.

Considering the predictive and prognostic analyses above, we identified ER, PR, AR and Ki67 as meaningful clinical indicators for HGSC molecular classification. A clustering analysis was performed to identify which tumors were related to each other according to ER, PR, AR and Ki67 expression. A total of five hormone receptor-based molecular subtypes were distinguished (PR+, PR − ER + AR+, PR − ER + AR−, PR − ER − AR+, and PR − ER − AR−). Ki67 did not play a dominant role in the classification (Fig. 1A,B).

Characteristics of patients within each subgroup are shown in Table 4. Patients in the PR+ group were younger compared to those in the other groups (p < 0.001). More patients were of advanced stage in the PR − ER + AR− group than in the other groups (p = 0.020). Patients in all subgroups received similar debulking surgery and adjuvant chemotherapy, and there was no difference in the surgery outcomes between the groups (p = 0.476). A greater proportion of patients were sensitive to platinum-based chemotherapy in the PR − ER − AR+ group compared with the other groups (p = 0.034).

The univariate Kaplan-Meier analysis for OS was performed (Fig. 1C), and a statistically significant trend of increasing risk of death was observed among the subgroups (χ^2^ = 16.140, p < 0.001, Table 5). In the multivariate analysis adjusted for age, FIGO stage and residual disease, patients also had orderly increased hazard ratios for death in the PR + (HR = 2.256, 0.983–5.175, p = 0.055), PR − ER + AR + (HR = 2.188, 1.004–4.796, p = 0.049), PR − ER − AR− (HR = 2.316, 1.097–5.082, p = 0.028) and PR − ER + AR− (HR = 2.928, 1.366–6.276, p = 0.006) subgroups compared with the PR − ER − AR+ subgroup (Table 5).

## Discussion

In this large mono-institutional study, six hormone receptors (ER, PR, AR, FSHR, LHR and GnRHR) and another two potentially useful biomarkers (HER2 and Ki67) were investigated. Based on the clustering expression patterns of four critical parameters (ER, PR, AR, and Ki67), we classified patients into five subgroups (PR+, PR − ER + AR+, PR − ER + AR−, PR − ER − AR+, and PR − ER − AR−) with distinctive clinic-pathological features.

Several studies have investigated the prognostic impact of hormone receptor expression in ovarian cancer. The majority of previous investigations focused on ER or PR expression levels, and these previous investigations obtained inconsistent results. Some studies implicated that ER or PR expression was associated with improved progression, while other studies showed no significant associations between ER or PR expression and prognosis^10^,^11^,^25^. Moreover, most studies combined all of the disease subtypes regardless of heterogeneity and had small sample sizes. This may hinder efforts to identify the subtype-specific significance of hormone receptor expression.

Sieh *et al.*^9^ first evaluated the prognostic effects of ER and PR expression according to histological subtypes. Their study showed that ER or PR was positive in the majority of HGSCs and endometroid ovarian carcinomas, while their expression was rare in clear cell or mucinous carcinomas. In the histological subgroup analyses, ER and PR expression were associated with improved survival in endometroid ovarian carcinoma, and PR expression was associated with better survival in HGSC. Our study focused on only one histological type HGSC, which is the most common subtype of epithelial ovarian cancer and has high expression levels of hormone receptors. In contrast to Sieh’s study, we used a cutoff value of 10% instead of 1% for ER− and PR−positive expression^19^. Studies of 875 HGSC patients show that ER expression is correlated with worse PFS and OS independently, while PR is only associated with improved PFS, but not OS. Our findings provide more information about the clinical significance of ER or PR expression in HGSC.

In addition, our study includes another promising biomarker, androgen receptor, which is seldom mentioned in ovarian cancer research^4^,^7^,^12^,^14^. Recent studies have highlighted androgen receptor as a promising prognostic and treatment-predictive marker of breast cancer^26^,^27^. Jonsson *et al.*^12^ demonstrated a favorable outcome for ovarian cancer patients whose tumors coexpressed PR and AR. Our study, for the first time, suggests that AR is an independent predictor of platinum sensitivity in HGSC and improved OS. The underlying mechanism of their relationship should be studied in the future.

Consequently, our study has provided us with the possibility to establish a molecular classification of HGSC based on hormone receptor expression. As another type of hormone-related cancers, breast cancers are classified into four subtypes based on corresponding ER, PR, HER2 and Ki67 expression levels^28^. Similar attempts have been made to stratify ovarian cancer according to ER, PR and HER2 expression, but no significant clinicopathological differences were observed between the subgroups^29^. According to previous studies, the frequencies of HER2 overexpression in ovarian cancer varied^30^. Possible explanations might be the use of different antibodies as well as variable scoring systems. We selected the Roche antibody identified by the FDA for the HER2 test and evaluated HER2 expression according to the ASCO/CAP guideline^23^. Our study indicates that HER2 overexpression is rare in HGSC, and thus the potential application of HER2 in HGSC classification is limited.

Based on our results, we classified HGSC patients into five subgroups according to ER, PR and AR expression. Kruchten *et al.*^7^ had also classified ovarian cancer according to ER, PR and AR expression. However, they combined all histological subtypes and did not refer to the hypothalamic-pituitary-gonadal axis-related hormone receptors. Our study included only patients diagnosed with HGSC, which excludes histological heterogeneity and allows for the identification of reliable and meaningful subgroups. Additionally, further investigation revealed that these groups have distinctive clinic-pathological characteristics. Only a small proportion of patients (4.3%) were PR − ER − AR+, which indicates a greater response to chemotherapy and improved OS. The proportion of the PR − ER + AR− subgroup ranked first (32.5%) in HGSC, and the prognosis of this group was the worst. Thus, those patients with worse prognosis might need more aggressive treatment and might require more frequent follow-up.

Our classifications indicate that we can predict patients’ clinical outcomes by routine assessment of ER, PR and AR expression. This could help guide individual treatment and stratification of patients in future clinical trials. Hormone therapy has long been an alternative therapy for breast cancer^31^. However, hormone therapy has only been regarded as a salvage therapy for ovarian cancer. Previous studies have shown that the effect of endocrine therapy could be influenced by hormone receptor status with subgroup heterogeneity^32^,^33^,^34^,^35^. Our hormone receptor-based classification might be used in the selection of potential patients for endocrine therapy in future studies.

Moreover, though not included in the molecular classification of HGSC, our investigations on other biomarkers provide us with more information about future precise treatments for HGSC patients. The data on FSHR, LHR, and GnRHR expression were scarce in previous studies, and our study found high expression levels of FSHR, LHR and GnRHR. This suggests the possibility of using corresponding ligands as targeting moieties^36^,^37^. For instance, Zhang *et al.*^36^ have developed a paclitaxel-loaded FSH binding peptide-targeted drug against ovarian cancer. Their *in vitro* and *in vivo* studies showed that this drug displays higher anti-tumor efficacy against FSHR-expressing tumors with less cytotoxicity. A phase II study of AEZ-108 (an LHRH agonist linked to doxorubicin) in LHRH-positive refractory ovarian cancer patients has been conducted^37^. Among treated patients, 14.3% (6/42) showed partial response and 38% (16/42) still had stable disease with a median PFS of 12 weeks and a median OS of 53 weeks. However, rare HER2 overexpression indicates the limited application potential of trastuzumab in HGSC.

In conclusion, for our study, we recruited a group of patients with the same histology who were treated within 9 years and underwent similar treatment procedures. We proposed an effective and clinically applicable classification of HGSC in light of our discovery. Indeed, it should also be further verified in an independent external cohort, and we think that a multicenter prospective cohort is preferred for this purpose. Furthermore, the underlying molecular pathogenesis mechanism of distinct subgroups should also be studied in the future. Although further investigations are necessary, our hormone receptor-based classification could help guide individual treatments and stratify patients in future studies.

## Additional Information

**How to cite this article**: Feng, Z. *et al.* A clinically applicable molecular classification for high-grade serous ovarian cancer based on hormone receptor expression. *Sci. Rep.* **6**, 25408; doi: 10.1038/srep25408 (2016).

## Supplementary Material

Supplementary Information

## Figures and Tables

**Table 1:** Characteristics of patients and hormone receptor expression (n = 875).

Age at diagnosis, median (range), years		56 (30–90)	
Follow-up time, median (range), months		29(1–115)	
Progression-free survival	2-year: 38.1%, 5-year: 19.4%		
Overall survival	2-year: 79.3%, 5-year: 48.8%		
Menopausal status	Postmenopausal	602	69%
	Premenopausal	273	31%
Family history (breast or ovarian cancer)	Yes	85	10%
	No	790	90%
ECOG	0	512	59%
	1	298	34%
	2	65	7%
FIGO	Early (FIGO I, II)	75	9%
	Advanced (FIGO III, IV)	800	91%
Residual Disease	R0	272	31%
	RD	603	69%
Platinum sensitivity	Yes	568	67%
	No	237	28%
	NA	44	5%
ER	Positive (>10%)	556	64%
	Negative (≤10%)	307	36%
PR	Positive (>10%)	109	13%
	Negative (≤10%)	755	87%
AR	Positive (>10%)	309	36%
	Negative (≤10%)	559	64%
FSHR	Positive (IRS ≥ 3)	470	55%
	Negative (IRS < 3)	393	46%
LHR	Positive (IRS ≥ 3)	301	35%
	Negative (IRS < 3)	563	65%
GnRHR	Negative	100	12%
	Weak	175	20%
	Moderate	306	36%
	Strong	276	32%
HER2	0	833	96%
	≥1	31	4%
Ki67	Positive (>50%)	223	26%
	Negative (≤50%)	644	74%

**Table 2:** Association between receptor expression and platinum sensitivity.

			Platinum sensitivity		P value
Parameters			Yes	No	
ER	Positive	514	365	149	0.685
			71.0%	29.0%	
	Negative	280	195	85	
			69.6%	30.4%	
PR	Positive	102	82	20	0.020
			80.4%	19.6%	
	Negative	692	478	214	
			69.1%	30.9%	
AR	Positive	290	221	69	0.010
			76.2%	23.8%	
	Negative	508	342	166	
			67.3%	32.7%	
FSHR	Positive	431	303	128	0.938
			70.3%	29.7%	
	Negative	363	257	106	
			70.8%	29.2%	
LHR	Positive	276	198	78	0.682
			71.7%	28.3%	
	Negative	518	363	155	
			70.1%	29.9%	
GnRHR	Positive	700	494	206	0.902
			70.6%	29.4%	
	Negative	89	62	27	
			69.7%	30.3%	
HER2	0	767	539	228	0.831
			70.3%	29.7%	
	≥1	27	20	7	
			74.1%	25.9%	
Ki67	Positive	206	159	47	0.016
			77.2%	22.8%	
	Negative	591	403	188	
			68.2%	31.8%	

**Table 3:** Univariate and multivariate analyses of factors associated with PFS and OS^1^.

Parameters	PFS							OS						
	Univariate^2^	Multivariate^3^						Univariate^2^	Multivariate^3^					
	P value	Referent	HR	95% CI			P value	P value	Referent	HR	95% CI			P value
Age	–	Continuous Variable	0.997	0.988	–	1.007	0.576	–	Continuous Variable	1.016	1.005	–	1.028	0.006
FIGO Stage	<0.001	Advanced *vs.* Early	2.413	1.577	–	3.691	<0.001	<0.001	Advanced *vs.* Early	3.618	1.728	–	7.576	0.001
Residual Disease	<0.001	RD *vs.* R0	1.633	1.324	–	2.015	<0.001	<0.001	RD *vs.* R0	1.958	1.457	–	2.631	<0.001
ER	0.036	Negative	1.302	1.077	–	1.573	0.006	0.150	Negative	1.288	1.014	–	1.636	0.038
PR	0.009	Negative	0.718	0.538	–	0.958	0.024	0.061	Negative	0.911	0.617	–	1.346	0.641
AR	0.368	Negative	0.982	0.813	–	1.185	0.847	0.023	Negative	0.744	0.578	–	0.959	0.022
FSHR	0.975	Negative	1.024	0.862	–	1.217	0.786	0.586	Negative	1.166	0.936	–	1.451	0.171
LHR	0.455	Negative	1.108	0.925	–	1.329	0.266	0.782	Negative	0.971	0.772	–	1.222	0.805
GnRHR	0.800	Negative	0.907	0.691	–	1.192	0.484	0.488	Negative	0.976	0.693	–	1.374	0.889
HER2	0.746	0	0.916	0.576	–	1.457	0.710	0.815	0	0.747	0.407	–	1.373	0.348
Ki67	0.021	Negative	0.835	0.683	–	1.021	0.079	0.003	Negative	0.688	0.520	–	0.910	0.009

^1^Numbers of patients (number of events/patients at risk): PFS (549/749), OS (345/875).

^2^Log-rank tests.

^3^Cox regression analysis expressed as hazard ratios.

**Table 4:** Characteristics of patients within subgroups.

Parameters		PR − ER − AR+		PR+		PR − ER + AR+		PR − ER − AR−		PR − ER + AR−		P value
Age at diagnosis, median (range), years		56(37–79)		50(31–90)		56(36–81)		55(30–82)		57(34–84)		<0.001
Menopausal status	Postmenopausal	23	63.9%	45	46.9%	131	70.1%	166	66.9%	211	77.3%	<0.001
	Premenopausal	13	36.1%	51	53.1%	56	29.9%	82	33.1%	62	22.7%	
Family history (breast or ovarian cancer)	Yes	3	8.3%	12	12.5%	20	10.7%	16	6.5%	30	11.0%	0.322
	No	33	91.7%	84	87.5%	167	89.3%	232	93.5%	243	89.0%	
ECOG	0	26	72.2%	66	68.8%	109	58.3%	146	58.9%	154	56.4%	0.117
	1	5	13.9%	25	26.0%	63	33.7%	87	35.1%	99	36.3%	
	2	5	13.9%	5	5.2%	15	8.0%	15	6.0%	20	7.3%	
FIGO	Early (FIGO I, II)	4	11.1%	16	16.7%	15	8.0%	22	8.9%	15	5.5%	0.020
	Advanced (FIGO III, IV)	32	88.9%	80	83.3%	172	92.0%	226	91.1%	258	94.5%	
Residual Disease	R0	12	33.3%	37	38.5%	58	31.0%	70	28.2%	85	31.1%	0.476
	RD	24	66.7%	59	61.5%	129	69.0%	178	71.8%	188	68.9%	
Platinum sensitivity	Yes	31	86.1%	71	76.3%	131	71.6%	149	62.6%	166	62.6%	0.034
	No	5	13.9%	19	20.4%	45	24.6%	74	31.1%	82	30.9%	
	NA	0	0.0%	3	3.2%	7	3.8%	15	6.3%	17	6.4%	

**Table 5:** Risk of death in patients with HGSC depending on subgroup classification.

Subgroup	N	%	Overall death	Trend test		Overall survival			
				P value	χ^2^	Adjusted HR (95% CI)	P value	2-year	5-year
PR − ER − AR+	36	4.3%	7(19.4%)	<0.001	16.140	Referent	–	97.1%	67.4%
PR+	96	11.4%	28(29.2%)			2.256(0.983–5.175)	0.055	87.3%	51.5%
PR − ER + AR+	187	22.3%	68(36.4%)			2.188(1.004–4.796)	0.049	83.7%	49.6%
PR − ER − AR−	248	29.5%	103(41.5%)			2.316(1.097–5.082)	0.028	77.7%	51.8%
PR − ER + AR−	273	32.5%	126(46.2%)			2.928(1.366–6.276)	0.006	75.1%	42.1%
