# Supplementary material for: A clinically applicable molecular classification for high-grade serous ovarian cancer based on hormone receptor expression
Source: Sci Rep. 2016 May 3;6:25408. doi: 10.1038/srep25408 (PMC4853732; doi:10.1038/srep25408)
Supplement: Supplementary Information [file srep25408-s1.doc]

**A clinically applicable molecular classification for high-grade serous ovarian cancer based on hormone receptor expression**

Zheng Feng^12*^, Hao Wen^12*^, Rui Bi^23*^, Xingzhu Ju^12^, Xiaojun Chen^12^, Wentao Yang^23^, Xiaohua Wu^12^

*These authors contributed equally to this work.

**
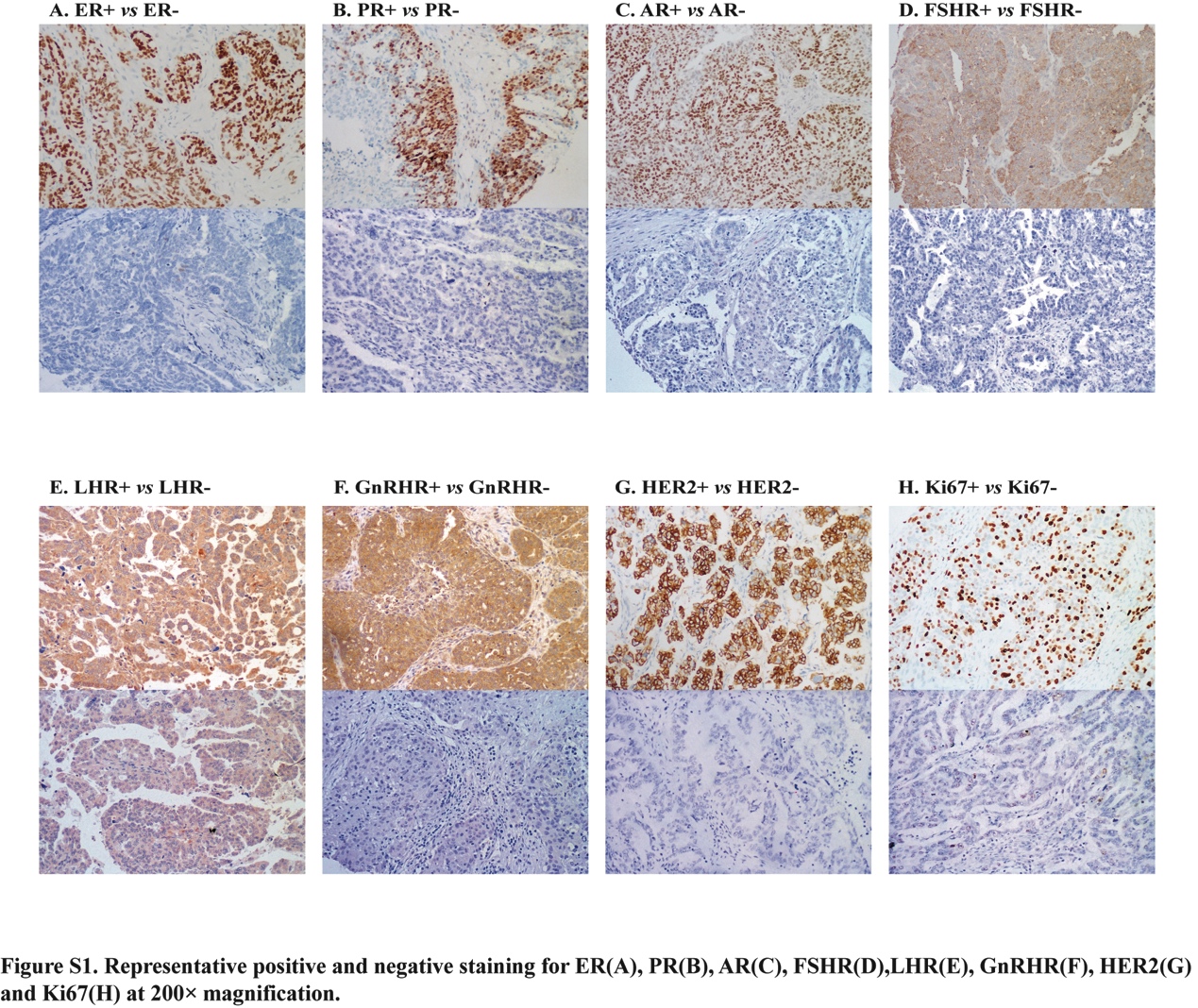
**

**
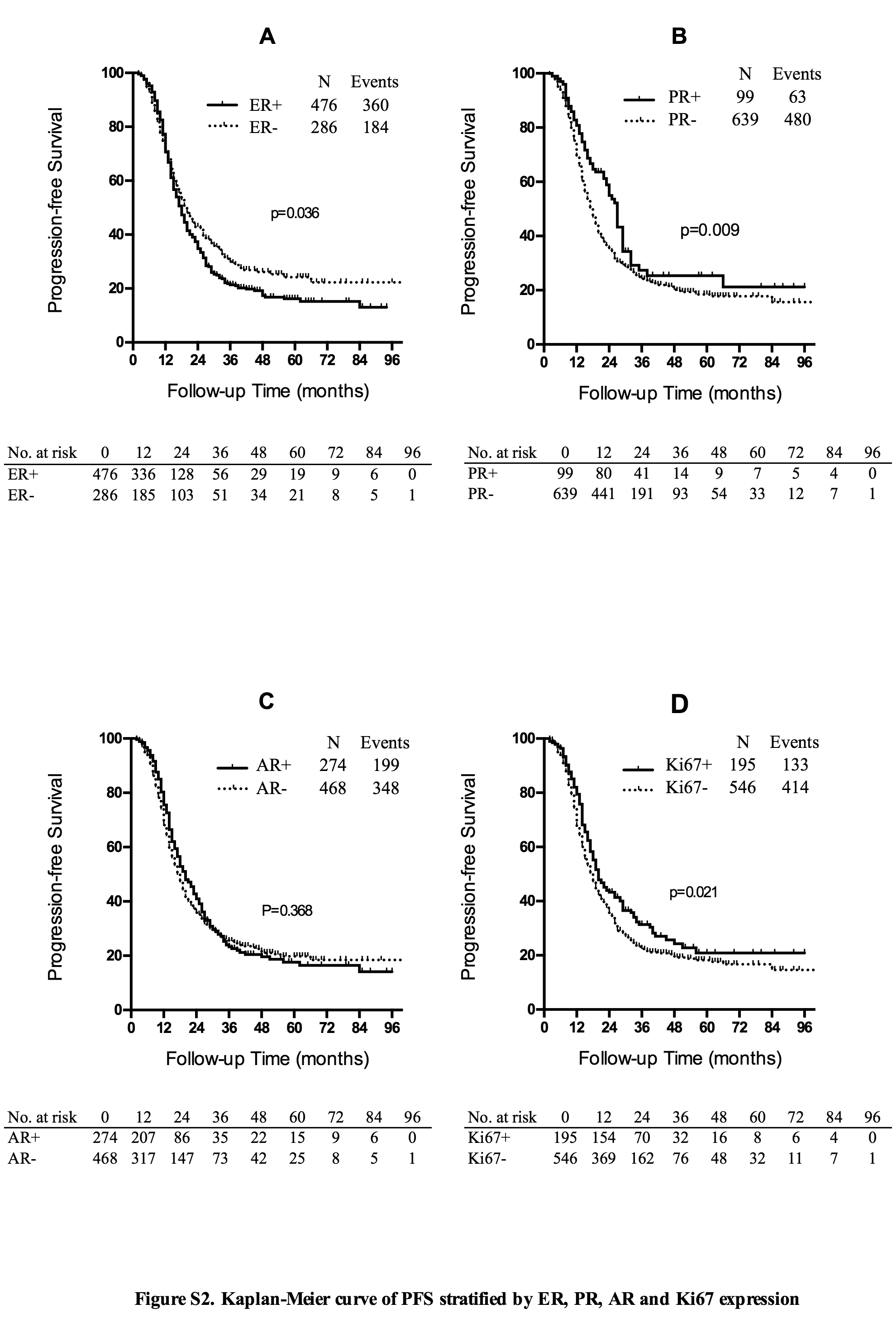
**

**
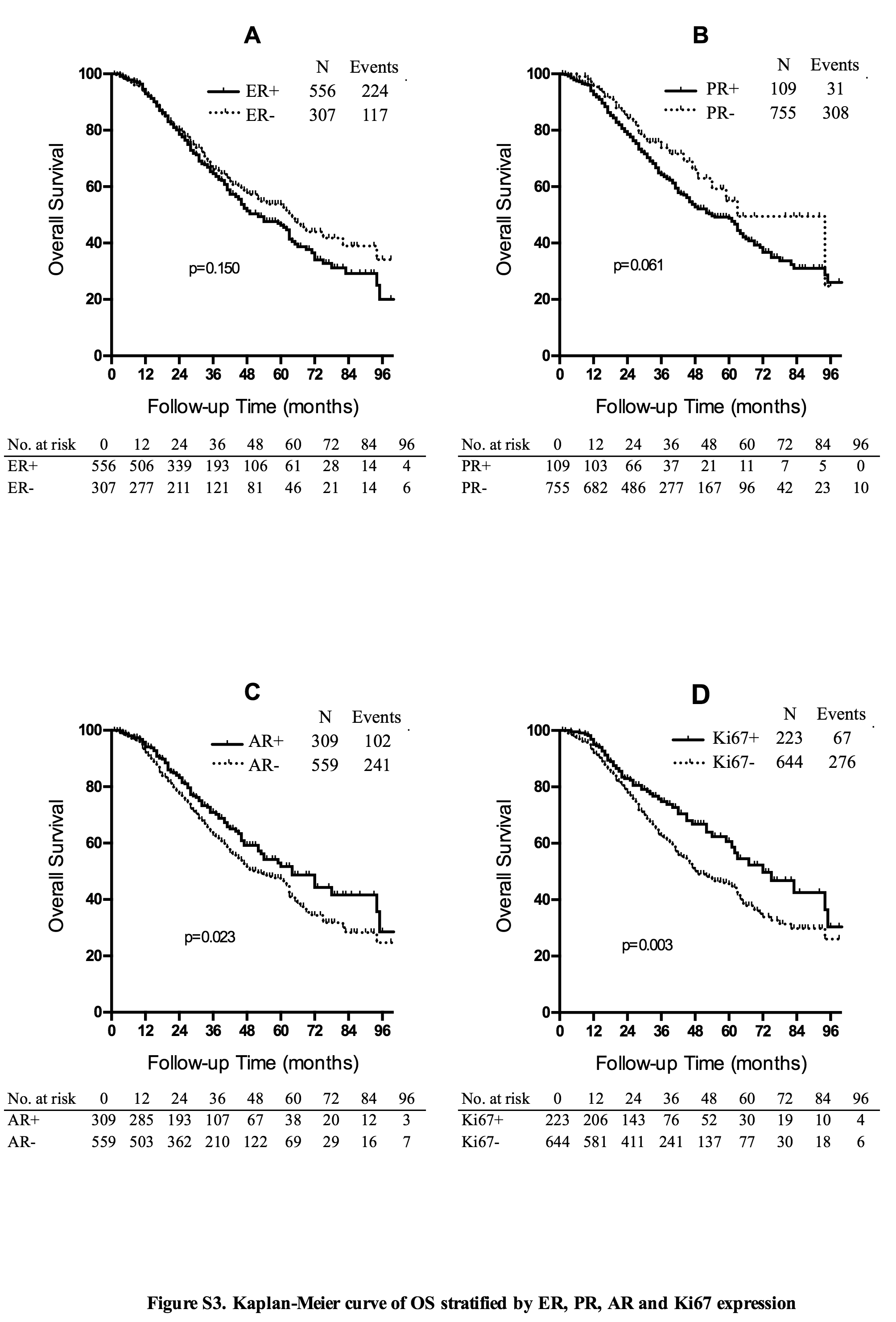
**

| **Table S1. Associations of parameter expression (p value)** | | | | | | | | | | | | | | | | | |
| --- | --- | --- | --- | --- | --- | --- | --- | --- | --- | --- | --- | --- | --- | --- | --- | --- | --- |
| Parameters | | ER | | PR | | AR | | FSHR | | LHR | | GnRHR | | HER2 | | Ki67 | |
|  |  | + | - | + | - | + | - | + | - | + | - | + | - | + | - | + | - |
| ER | + | - | |  | |  | |  | |  | |  | |  | |  | |
|  | - |  |  |  |  |  |  |  |  |  |  |  |  |  |  |  |  |
| PR | + | 87 | 469^1^ | - | |  | |  | |  | |  | |  | |  | |
|  | - | 22 | 283 |  |  |  |  |  |  |  |  |  |  |  |  |  |  |
| AR | + | 257 | 299^2^ | 76 | 233^3^ | - | |  | |  | |  | |  | |  | |
|  | - | 52 | 255 | 33 | 522 |  |  |  |  |  |  |  |  |  |  |  |  |
| FSHR | + | NS | | NS | | 186 | 283^6^ | - | |  | |  | |  | |  | |
|  | - |  |  |  |  | 123 | 270 |  |  |  |  |  |  |  |  |  |  |
| LHR | + | NS | | NS | | 86 | 215^7^ | NS | | - | |  | |  | |  | |
|  | - |  |  |  |  | 220 | 342 |  |  |  |  |  |  |  |  |  |  |
| GnRHR | + | NS | | 80 | 673^4^ | NS | | NS | | 274 | 480^9^ | - | |  | |  | |
|  | - |  |  | 23 | 76 |  |  |  |  | 26 | 75 |  |  |  |  |  |  |
| HER2 | + | NS | | NS | | NS | | NS | | NS | | NS | | - | |  | |
|  | - |  |  |  |  |  |  |  |  |  |  |  |  |  |  |  |  |
| Ki67 | + | NS | | 37 | 185^5^ | NS | | 136 | 86^8^ | NS | | NS | | NS | | - | |
|  | - |  |  | 72 | 569 |  |  | 333 | 307 |  |  |  |  |  |  |  |  |

1. NS: Not Significant.

2. P value: P_1_<0.001, P_2_<0.001, P_3_<0.001, P_4_=0.001, P_5_=0.046, P_6_=0.013, P_7_=0.002, P_8_=0.019, P_9_=0.026

| **Table S2. Association between receptor expression and residual disease** | | | | | |
| --- | --- | --- | --- | --- | --- |
| Parameters | | | Residual disease | | P value |
|  |  |  | R0 | RD |  |
| ER | Positive | 556 | 176 | 380 | 0.590 |
|  |  |  | 31.7% | 68.3% |  |
|  | Negative | 307 | 91 | 216 |  |
|  |  |  | 29.6% | 70.4% |  |
| PR | Positive | 109 | 41 | 68 | 0.120 |
|  |  |  | 37.6% | 62.4% |  |
|  | Negative | 755 | 226 | 529 |  |
|  |  |  | 29.9% | 70.1% |  |
| AR | Positive | 309 | 104 | 205 | 0.193 |
|  |  |  | 33.7% | 66.3% |  |
|  | Negative | 559 | 164 | 395 |  |
|  |  |  | 29.3% | 70.7% |  |
| FSHR | Positive | 470 | 154 | 316 | 0.210 |
|  |  |  | 32.8% | 67.2% |  |
|  | Negative | 393 | 113 | 280 |  |
|  |  |  | 28.8% | 71.2% |  |
| LHR | Positive | 301 | 86 | 215 | 0.315 |
|  |  |  | 28.6% | 71.4% |  |
|  | Negative | 563 | 181 | 382 |  |
|  |  |  | 32.1% | 67.9% |  |
| GnRHR | Positive | 757 | 233 | 524 | 0.818 |
|  |  |  | 30.8% | 69.2% |  |
|  | Negative | 100 | 32 | 68 |  |
|  |  |  | 32.0% | 68.0% |  |
| HER2 | 0 | 833 | 258 | 575 | 0.692 |
|  |  |  | 31.0% | 69.0% |  |
|  | ≥1 | 31 | 8 | 23 |  |
|  |  |  | 25.8% | 74.2% |  |
| Ki67 | Positive | 223 | 76 | 147 | 0.240 |
|  |  |  | 34.1% | 65.9% |  |
|  | Negative | 644 | 192 | 452 |  |
|  |  |  | 29.8% | 70.2% |  |

| **Table S3. Multivariate analysis of all receptor expression associated with platinum sensitivity*** | | | | | | |
| --- | --- | --- | --- | --- | --- | --- |
| Parameters | Referent | OR | 95%CI | | | P value |
| ER | Negative | 1.067 | 0.759 | - | 1.501 | 0.710 |
| PR | Negative | 0.679 | 0.390 | - | 1.180 | 0.170 |
| AR | Negative | 0.625 | 0.434 | - | 0.900 | 0.011 |
| FSHR | Negative | 1.158 | 0.842 | - | 1.593 | 0.366 |
| LHR | Negative | 0.910 | 0.652 | - | 1.272 | 0.582 |
| GnRHR | Negative | 0.956 | 0.580 | - | 1.574 | 0.859 |
| HER2 | 0 | 0.866 | 0.356 | - | 2.105 | 0.751 |
| Ki67 | Negative | 0.632 | 0.429 | - | 0.931 | 0.020 |

*Logistic regression analysis including all hormone receptor expression data in 568 platinum sensitive patients *vs* 237 platinum resistant patients.

| **Table S4. Risk of death depending on parameter status** | | | | |
| --- | --- | --- | --- | --- |
| Parameters | Status | Patients at risk | Numbers of events | Overall death (%) |
| ER | Positive (>10%) | 556 | 224 | 40.3% |
|  | Negative (<10%) | 307 | 117 | 38.1% |
| PR | Positive (>10%) | 109 | 31 | 28.4% |
|  | Negative (<10%) | 755 | 308 | 40.8% |
| AR | Positive (>10%) | 309 | 102 | 33.0% |
|  | Negative (<10%) | 559 | 241 | 43.1% |
| FSHR | Positive (IRS>3) | 470 | 177 | 37.7% |
|  | Negative (IRS<3) | 393 | 164 | 41.7% |
| LHR | Positive (IRS>3) | 301 | 124 | 41.2% |
|  | Negative (IRS<3) | 563 | 217 | 38.5% |
| GnRHR | Negative | 100 | 40 | 40.0% |
|  | Weak | 175 | 61 | 34.9% |
|  | Moderate | 306 | 130 | 42.5% |
|  | Strong | 276 | 110 | 39.9% |
| HER2 | 0 | 833 | 331 | 39.7% |
|  | >1 | 31 | 11 | 35.5% |
| Ki67 | Positive (>50%) | 223 | 67 | 30.0% |
|  | Negative (<50%) | 644 | 276 | 42.9% |
